# Alcohol and Tea Consumption in Relation to Liver Cancer Risk by Diabetes Status: A Prospective Cohort Study of 0.5 Million Chinese Adults

**DOI:** 10.3390/nu17172870

**Published:** 2025-09-04

**Authors:** Xiaoru Feng, Ruoqian Li, Minqing Yan, Changzheng Yuan, You Wu

**Affiliations:** 1 School of Biomedical Engineering, Tsinghua Medicine, Tsinghua University, Beijing 100084, China; 2 School of Healthcare Management, Tsinghua Medicine, Tsinghua University, Beijing 100084, China; 3 School of Public Health, Zhejiang University School of Medicine, Hangzhou 310058, China; 4 School of Basic Medical Sciences, Tsinghua Medicine, Tsinghua University, Beijing 100084, China; 5 Department of Health Policy and Management, Bloomberg School of Public Health, Johns Hopkins University, Baltimore, MD 21205, USA

**Keywords:** alcohol consumption, tea consumption, diabetes, liver cancer, prospective cohort study, China Kadoorie Biobank

## Abstract

**Background:** Liver cancer is a significant disease burden, with metabolic factors potentially influencing its risk. Diabetics, due to metabolic abnormalities, may be more sensitive to environmental exposures. Beverages like tea and alcohol could impact liver cancer risk and may influence prevention in diabetics. **Methods**: This study included 30,289 diabetics and 482,292 non-diabetics aged 30–79 years from the China Kadoorie Biobank. Baseline alcohol and tea consumption during the past year was collected through questionnaires, including frequency, amount, duration, and types. Incident liver cancer cases were identified from the national health insurance system and local disease registries. Cox proportional hazards regression models estimated hazard ratios (HRs) and 95% confidence intervals (CIs). **Results**: During a median follow-up of 9.6 years for diabetics and 10.1 years for non-diabetics, 193 (0.69 cases/1000 person-years) and 398 (0.45 cases/1000 person-years) incident liver cancer cases were documented, respectively. Weekly alcohol consumption was associated with higher liver cancer risk in both groups, stronger in diabetics (HR = 1.62; 95% CI: 1.12, 2.34) than in non-diabetics (HR = 1.20, 95% CI: 1.07, 1.35). Among diabetics, the risk was higher in some weekly alcohol consumption subgroups: high-level intake (HR = 2.21; 95% CI: 1.28, 3.80), ≥30 years (HR = 1.70; 95% CI: 1.06, 2.71), or spirit (≥50% alcohol) alcohol-specific consumption (HR = 1.91; 95% CI: 1.20, 3.04), and these associations were stronger than those in non-diabetics. For weekly tea consumption, low-level intake (HR = 0.82; 95% CI: 0.68, 0.99), <10 years (HR = 0.74; 95% CI: 0.58, 0.93), 10–29 years (HR = 0.84; 95% CI: 0.71, 0.99), and green tea-specific consumption (HR = 0.86; 95% CI: 0.75, 0.98) were associated with reduced liver cancer risk in non-diabetics. However, these associations were not significant in those with diabetes. **Conclusions**: Weekly alcohol consumption is significantly associated with an increased risk of liver cancer, especially in diabetics, while tea consumption appears to lower risk only in non-diabetics, highlighting the need for alcohol reduction in diabetics.

## 1. Introduction

Liver cancer ranks as the sixth most common cancer worldwide and has become an important global public health challenge. Its burden shows obvious geographic disparities, with China alone accounting for more than 50% of global cases [1]. Diabetes, which results from metabolic disorders, affects multiple organ systems and can lead to various complications [2]. Notably, diabetes has been identified as a significant risk factor for liver cancer, increasing its incidence by more than twofold [3,4,5].

China’s deep-rooted alcohol and tea culture has made both beverages highly popular, and the corresponding associations with liver cancer or diabetes have been investigated but remains controversial or unclear. Some studies suggested that low-level alcohol consumption may reduce the risk of liver cancer [6], while others argued that excessive drinking increased the risk [7,8,9]. Some studies indicated that tea consumption may be a protective factor against liver cancer [10,11], while others found no significant association between tea consumption and liver cancer [12,13]. On the other hand, previous studies have shown that alcohol and tea consumption play a significant role in the development of diabetes and its complications. A U-shaped or J-shaped association has been observed between alcohol consumption and the risk of diabetes and its cardiovascular complications [14]. This pattern arose because low-level alcohol consumption has been associated with reduced insulin resistance, whereas excessive drinking can lead to pancreatic β-cell apoptosis [15,16]. It was also found that tea consumption might have a protective effect against type 2 diabetes and its microvascular complications [17,18], attributed to the bioactive substances in tea, particularly tea polyphenols [19,20]. Based on the above evidence, individuals with diabetes may be more sensitive to the consumption of tea and alcohol due to metabolic abnormalities, which may play a role in liver cancer risk. However, critical gaps persist regarding the relationship between beverage consumption and liver cancer secondary to diabetes, as liver cancer is one of the major complications of diabetes. Despite mechanistic plausibility, only a few small-sample studies have addressed this topic and were limited to Western populations [21,22]. Large-scale prospective studies in the Chinese context are needed.

Based on 0.5 million Chinese adults from the China Kadoorie Biobank (CKB), we aimed to investigate the association between alcohol and tea consumption and liver cancer and to assess whether it differs by diabetes status.

## 2. Materials and Methods

### 2.1. Study Population

The study design and participant characteristics of the CKB have been described previously [23,24,25]. In brief, between 2004 and 2008, the CKB project recruited 512,724 participants aged 30 to 79 years from 10 regions across China (5 urban and 5 rural areas). After obtaining written informed consent, trained investigators conducted questionnaire interviews, physical examinations, and biological sample collection. Every 4–5 years, a resurvey is conducted on a randomly selected 5% subset of the cohort. The study was approved by the ethics committees of the Chinese Center for Disease Control and Prevention and the University of Oxford.

To analyze the association between beverage consumption and the risk of liver cancer secondary to diabetes (Figure S1), we first excluded anomalous cases (*n* = 106), including participants with negative values for follow-up duration (*n* = 1), years of alcohol consumption (*n* = 26) or years of tea drinking (*n* = 79) at baseline. Additionally, we excluded participants diagnosed with liver cancer at baseline (*n* = 37). The final analysis included 512,581 participants, comprising 30,289 individuals with diabetes and 482,292 without diabetes.

### 2.2. Assessment of Alcohol and Tea Consumption

In the baseline survey, all participants were asked to report their alcohol consumption frequency during the past 12 months, categorized as never, occasional, seasonal, monthly but less than weekly, or weekly. Those who drank alcohol weekly were further asked to provide details on the following: (1) days consuming in a typical week (1–2 days, 3–5 days, or almost every day); (2) grams of alcohol consumed on a typical drinking day; (3) age when they started drinking weekly; and (4) type of alcohol consumed most commonly (beer, rice wine, wine, spirits with <50% alcohol, or spirits with ≥50% alcohol). The total grams of alcohol consumed weekly were calculated by multiplying drinking days per week by grams per drinking day. For this analysis, participants were categorized into two groups based on alcohol consumption frequency: less than weekly, and weekly. Weekly consumers were further classified according to weekly alcohol consumption (low: male ≤ 110 g/week, female ≤ 30 g/week; moderate: male 110– 407 g/week, female 30– 135 g/week; high: male >407 g/week, female >135 g/week), duration of alcohol consumption (<10 years, 10–29 years, and ≥30 years), and types of alcohol consumed.

Similar to collecting information about alcohol consumption, all participants were asked to report their tea consumption frequency during the past 12 months, categorized as never, occasional, seasonal, monthly but less than weekly, or weekly. Those who drank tea weekly were further asked to provide details on the following: (1) days consuming in a typical week (1–2 days, 3–5 days, or almost every day); (2) times of changing tea leaves on a typical consuming day; (3) grams of tea leaves added each time; (4) age when they started drinking weekly; and (5) type of tea consumed most commonly (green tea, oolong tea, black tea, or others). The grams of tea consumed on a typical consuming day were calculated as the following: Tea grams per day = (Number of leaf changes per day + 1) × Grams added per change, and total grams of tea consumed weekly were calculated as the following: Tea grams per week = Tea grams per day × Number of consuming days per week. For example, if a participant changed tea leaves 3 times per day, added 2 g each time, and consumed tea 5 days per week, the daily consumption would be 8 g and the weekly consumption 40 g. For this analysis, participants were categorized into three groups based on tea consumption frequency: never, less than weekly, and weekly. Weekly consumers were further classified according to weekly tea consumption (low: ≤12 g/week; moderate: 12–26 g/week; high: >26 g/week), duration of tea consumption (<10 years, 10–29 years, and ≥30 years), and types of tea consumed (green tea and others).

### 2.3. Assessment of Covariates

The covariate information was derived from the baseline questionnaire, which included sociodemographic characteristics (age, sex, region, education, marital status, and household income), lifestyle behaviors (smoking, physical activity, BMI, and intake of meat, fruit and vegetables), medical history (diabetes, hypertension, chronic liver diseases such as chronic hepatitis, cirrhosis and hepatitis B), and family history (parents or siblings with cancer or diabetes). Habitual dietary intake over the past year was assessed using a qualitative food frequency questionnaire. Daily physical activity level was quantified by summing the metabolic equivalent of task (MET) hours for all activities, using the hours spent on each activity as a weight. Body height and weight were measured at baseline by trained staff using uniformly calibrated instruments, and BMI was calculated by dividing weight by the square of height. Prevalent diabetes was defined as measured fasting blood glucose ≥ 7.0 mmol/L, measured random blood glucose ≥ 11.1 mmol/L, or self-reported diagnosis of diabetes.

### 2.4. Ascertainment of Liver Cancer Cases

The vital status of all CKB participants was obtained by linking their unique national identification number with the national health insurance claim databases, local disease surveillance points system, disease and death registries. Participants who failed to be linked to the health insurance database were actively followed annually by staff to determine their status. The trained staff who were blinded to the baseline information used the International Classification of Diseases, 10th Revision (ICD-10), to code all disease diagnoses. The endpoint outcome defined in this analysis was the incidence of liver cancer (C22).

### 2.5. Statistical Analysis

We described the baseline characteristics of participants using mean ± standard deviation (SD) or number (*n*) with percentage (%). To enhance comparability between the diabetic and non-diabetic groups, we performed 1:3 propensity score matching (PSM) on age and sex for baseline characteristics. To compare characteristics across different frequency groups of alcohol and tea consumption, we applied Pearson’s Chi-squared test, Fisher’s exact test, Welch’s two-sample *t*-test, or ANOVA, as appropriate. Follow-up time (person years) was calculated from the baseline date to the date of the outcome event, death, loss to follow-up, or 31 December 2016 (the end of the follow-up period in this study), whichever occurred first.

We used Cox proportional hazards models to estimate the hazard ratios (HRs) and 95% confidence intervals (CIs) for the association between alcohol or tea consumption and liver cancer risk in individuals with and without diabetes, separately. The proportional hazard assumption for the Cox regression models was tested using the Schoenfeld residual and no violation was discovered. Participants who consumed alcohol less than weekly or never consumed tea during the past 12 months served as the reference group. We examined the associations between liver cancer incidence and both the frequency and weekly amount of alcohol or tea consumption. Additionally, we quantified the associations between liver cancer risk and the duration of alcohol or tea consumption, as well as the types of alcohol or tea consumed, by comparing weekly consumers with the reference group. All models were adjusted for age, sex, region, education, marital status, household income, smoking, meat intake, vegetable intake, fruit intake, physical activity, BMI, chronic liver disease, hypertension, and family history. Linear trend tests were only performed in weekly alcohol or tea consumers, by assigning the median value of weekly alcohol or tea consumption and the duration of alcohol or tea consumption to each category and including them as continuous variables in models. Relative excess risk due to interaction (RERI), proportion attributable to interaction (AP), and synergy index (S) were applied to evaluate interactions [26]. Dose–response curves were depicted using restricted cubic splines (RCS), and cumulative incidence over time was estimated with Kaplan–Meier curves.

Further, among the diabetic participants, we conducted subgroup analyses according to baseline characteristics (age, sex, region, smoking, physical activity, BMI) and performed interaction tests using likelihood ratio tests. To assess the robustness of our results, we also conducted the following sensitivity analyses within the diabetic participants: (1) excluding participants who were diagnosed with liver cancer during the first two years of follow-up; (2) excluding participants with any cancer, chronic heart disease, stroke or transient ischemic attack, gallstones or gallbladder disease, or kidney disease at baseline; (3) incorporating regional HBV prevalence from the Fourth National Serological Survey as an instrumental contextual covariate in the model [27].

All statistical analyses were performed with R software version 4.2.2 (31 October 2022; R Foundation for Statistical Computing). All *p*-values were two-sided, and statistical significance was defined as *p* < 0.05.

## 3. Results

### 3.1. Characteristics of Participants

Diabetic participants included in analyses had a mean age of 58.19 ± 9.58 years, with 38.56% being male, while non-diabetic participants had a mean age of 51.64 ± 10.63 years, with 41.14% being male. The proportion of weekly alcohol drinkers was 12.35% among diabetic participants and 13.89% among non-diabetic participants (Table 1). In both diabetic and non-diabetic groups, weekly alcohol drinkers were more likely to be younger, male, and smokers compared with those who drank less than weekly.

Similarly, the proportion of weekly tea drinkers was 31.43% among diabetic participants and 32.51% among non-diabetic participants (Table 2). Compared with those who never drank tea, weekly tea drinkers were more likely to be younger, male, and smokers. The correlation between alcohol and tea consumption frequency was weakly positive in this population (Spearman’s *ρ* = 0.228, *p* < 0.001).

### 3.2. Risk of Liver Cancer by Frequency and Amount of Beverage Consumption

Among participants with diabetes, during a median follow-up of 9.6 years (282,227 person-years in total), we identified 193 incident liver cancer cases, with a crude incidence rate of 0.68 cases/1000 person-years. Among participants without diabetes, during a median follow-up of 10.1 years (887,098 person-years in total), 398 incident liver cancer cases were identified, with a crude incidence rate of 0.45 cases/1000 person-years. Kaplan–Meier curves of liver cancer incidence stratified by alcohol and tea consumption in participants with and without diabetes are shown in Figure S2.

**Figure 1:**
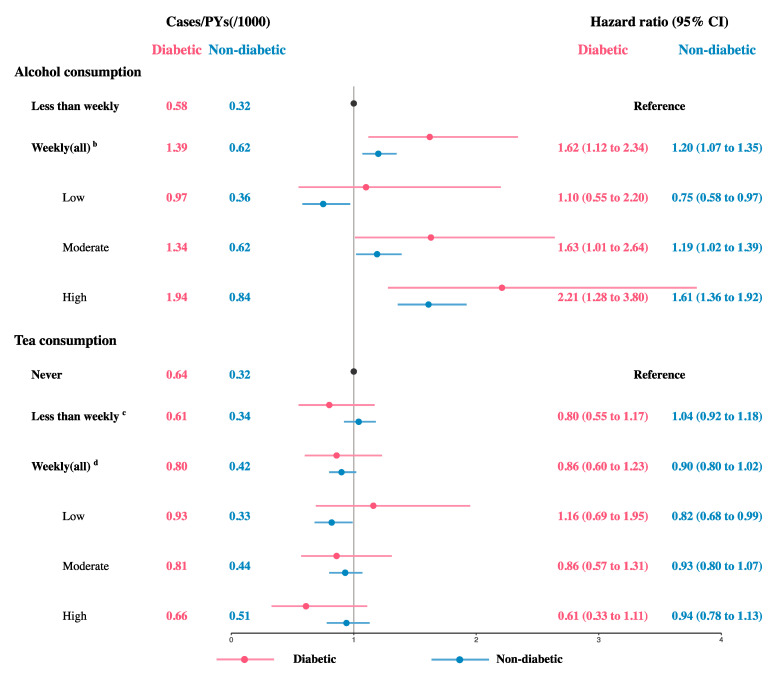
Hazard ratios for liver cancer incidence according to frequency and amount of beverage consumption (*n* = 512,581) ^a^. Abbreviations: CI, confidence interval; PYs, person years. ^a^ Adjusted for age, sex, region, education, marital status, household income, smoking, meat intake, vegetable intake, fruit intake, physical activity, BMI, chronic liver disease, hypertension, and family history. ^b^ Classified by quartiles: low (male: ≤110 g/week; female: ≤30 g/week); moderate (male: 110–407 g/week; female: 30–135 g/week); high (male: >407 g/week; female: >135 g/week). ^c^ Those who never drink tea were not included. ^d^ Classified by quartiles: low (≤12 g/week); moderate (12–26 g/week); high (>26 g/week).

After multivariable adjustment, among participants with diabetes, the HR (95% CI) for weekly drinkers compared with those who drank less than weekly was 1.62 (1.12, 2.34) (Figure 1). Specifically, for moderate and high levels of weekly alcohol consumption, the HRs (95% CIs) were 1.63 (1.01, 2.64) and 2.21 (1.28, 3.80), respectively. However, we did not observe a further increase in risk with higher alcohol consumption (*p*-trend = 0.211). Among participants without diabetes, compared with those who drank less than weekly, the HR (95% CI) for weekly drinkers was 1.20 (1.07, 1.35). For low, moderate, and high levels of weekly alcohol consumption, the HRs (95% CIs) were 0.75 (0.58, 0.97), 1.19 (1.02, 1.39), and 1.61 (1.36, 1.92), respectively, and the risk increased with higher alcohol consumption (*p*-trend < 0.01). In addition, diabetes and weekly alcohol consumption demonstrated positive additive interaction (RERI > 0, AP > 0, S > 1) (Table S1).

Among participants with diabetes, tea consumption frequency was not associated with risk of liver cancer incidence (Figure 1). However, we observed a reduction in liver cancer risk with increasing weekly tea consumption (*p*-trend = 0.015). Among participants without diabetes, compared with those who never drank tea, the HR (95% CI) for weekly tea consumers with low consumption was 0.82 (0.68, 0.99). However, we did not observe a further reduction in risk with higher tea consumption (*p*-trend = 0.436). Diabetes and no tea consumption also showed positive additive interaction (RERI > 0, AP > 0, S > 1) (Table S1).

### 3.3. Risk of Liver Cancer by Duration and Type of Beverage Consumption

We further analyzed the association between the duration and types of alcohol and tea consumption and liver cancer incidence. Among participants with diabetes, an obvious increase in risk of liver cancer was seen in weekly alcohol consumers who consumed alcohol for ≥30 years (HR = 1.70; 95% CI: 1.06, 2.71) or who consumed high-proof spirits (≥50% alcohol) (HR = 1.91; 95% CI: 1.20, 3.04) compared with those who drank less than weekly (Table 3). However, the risk of liver cancer did not increase with longer alcohol consumption duration (*p*-trend = 0.752). Similarly, among participants without diabetes, weekly alcohol consumers who consumed alcohol for ≥30 years (HR = 1.35; 95% CI: 1.16, 1.58), or who consumed high-proof spirits (≥50% alcohol) (HR = 1.24; 95% CI: 1.07, 1.45) or low-proof spirits (<50% alcohol) (HR = 1.42; 95% CI: 1.17, 1.72) had a higher risk. However, the risk of liver cancer did not increase with longer alcohol consumption duration (*p*-trend = 0.081).

In diabetic participants, neither the duration nor types of tea consumption were associated with liver cancer incidence, and the risk of liver cancer did not decrease with increasing tea consumption duration (*p*-trend = 0.515). In non-diabetic participants, compared with those who never drank tea, a decreased risk of liver cancer was observed in weekly tea consumers who drank tea for <10 years (HR = 0.74; 95% CI: 0.58, 0.93) or for 10–29 years (HR = 0.84; 95% CI: 0.71, 0.99). And the protective effect of tea consumption on liver cancer incidence diminished with increasing tea consumption duration (*p*-trend = 0.046). Additionally, weekly tea consumers who consumed green tea (HR = 0.86; 95% CI: 0.75, 0.98) had a lower risk compared with those who never drank tea. According to the dose–response curve, green tea intake and duration showed a trend of initially decreasing and then increasing liver cancer risk (Figure S3).

### 3.4. Subgroup Analyses

In the subgroup analyses of the diabetic participants, compared with those who drank alcohol less than weekly, weekly consumers exhibited a higher risk of liver cancer in the following subgroups: age 55–65 years (HR = 1.88; 95% CI: 1.08, 3.26), male (HR = 1.50; 95% CI: 1.02, 2.19), rural region (HR = 1.98; 95% CI: 1.08, 3.63), smoker (HR = 1.53; 95% CI: 1.04, 2.24), and BMI ≥ 25.0 kg/m^2^ (HR = 1.97; 95% CI: 1.16, 3.35) (Table S2). However, the associations were consistent across all subgroups stratified by potential baseline risk factors (*p*-interaction > 0.05).

Compared with those who never drank tea, weekly tea consumers exhibited a lower risk of liver cancer only among females (HR = 0.36; 95% CI: 0.16, 0.81; *p*-interaction by sex = 0.011) and never smokers (HR = 0.49; 95% CI: 0.25, 0.97; *p*-interaction by smoking status = 0.055) (Table S3).

### 3.5. Sensitivity Analyses

Sensitivity analyses showed no substantial changes in the results after excluding participants diagnosed with liver cancer in the first two years, excluding those with baseline comorbidities, or including regional HBV prevalence as a covariate (Tables S4 and S5).

## 4. Discussion

In this large prospective Chinese cohort, we observed distinct associations between tea/alcohol consumption and liver cancer risk, with notable differences between diabetic and non-diabetic populations. Alcohol consumption was associated with an increased risk of liver cancer, especially for the diabetic population. Compared with those who drank less than weekly during the past 12 months, diabetic patients who are weekly alcohol consumers had a 62% higher risk of liver cancer. The risk increased to 70% for weekly alcohol consumers who consumed alcohol for ≥30 years, and to 91% for those who consumed spirits (≥50% alcohol). The adverse effects of weekly alcohol consumption were particularly pronounced among diabetic participants aged 55–65 years, males, rural residents, smokers, or those who were overweight. Additionally, among participants who drank alcohol weekly, those with diabetes faced a 1.35-fold higher risk of liver cancer compared with those without diabetes. As for tea intake, although among non-diabetic participants, modest consumption was associated with reduced risk of liver cancer compared with no consumption, this effect was not observed in diabetic participants. However, among diabetic participants, weekly tea consumption was associated with a 64% reduced risk of liver cancer in females and a 51% reduced risk in never smokers. Overall, low alcohol and tea consumption was beneficial for non-diabetic participants, whereas this protective effect is absent in those with diabetes. High, frequent, or long-term alcohol consumption is harmful across the population, with an even greater risk observed among participants with diabetes.

Consistent with numerous previous studies, frequent, excessive, or prolonged alcohol consumption has been shown to increase the risk of liver cancer [7,8,9]. For example, a meta-analysis of 19 cohort studies found that compared with those who never drank alcohol, those consuming ≥3 drinks and ≥6 drinks per day had a significantly increased risk of liver cancer by 16% and 22%, respectively [9]. Several potential biological mechanisms have been proposed to explain the impact of alcohol on hepatocarcinogenesis, including the interference of acetaldehyde (the first metabolite of ethanol oxidation) with DNA synthesis and repair, increased reactive oxygen-species production and lipid peroxidation, and excessive hepatocyte regeneration due to the reduction in retinoid metabolism [16]. Furthermore, substantial evidence has established diabetes as a risk factor for liver cancer [3,4,5]. Insulin resistance and elevated levels of insulin-like growth factor I (IGF-I) may stimulate hepatocyte cell proliferation and inhibit apoptosis in the liver [28].

However, evidence associated between alcohol consumption and liver cancer secondary to diabetes remains limited and primarily focuses on Western populations. A prospective cohort study in the United States, involving 1,518,741 participants, reported that compared with those who never consumed alcohol, diabetic participants who consumed >5 drinks per day had a higher risk of hepatocellular carcinoma (HCC) (HR = 1.87; 95% CI: 1.21, 2.89) than non-diabetic participants (HR = 1.33; 95% CI: 1.00, 1.78) [21]. Similarly, a case–control study in California, which included 640 cases, found that compared with those who consumed ≤4 drinks per day, diabetic participants who consumed >4 drinks per day had a higher risk of HCC (HR = 17.3; 95% CI: 3.9, 77.6) than non-diabetic participants (HR = 3.4; 95% CI: 2.2, 5.4) [22]. These findings are generally consistent with our results, indicating that frequent and excessive alcohol consumption increases the risk of liver cancer secondary to diabetes. This may be attributed to alcohol-induced oxidative stress, which increases the susceptibility of diabetic patients to liver cirrhosis and promotes the development of liver cancer [29]. Our study provides novel evidence from a large Chinese cohort, complementing and extending the findings from Western populations.

The association between low level of alcohol consumption and liver cancer remains controversial. A meta-analysis including seven cohort studies and 13 case–control studies found no association between light drinking (≤1 drink per day) and liver cancer (HR = 1.03; 95% CI: 0.90, 1.17) [6]. Another meta-analysis involving 16 cohort studies showed that moderate drinking (<3 drinks per day) reduced the risk of liver cancer among non-Asian populations (HR = 0.66; 95% CI: 0.52, 0.84) [9]. In contrast to these meta-analyses, our study observed a potential reverse association between low-level weekly alcohol consumption and liver cancer incidence in non-diabetic participants, while this association was absent in diabetic participants. This difference suggested that diabetes might alter the underlying mechanism. In non-diabetic populations, low-level alcohol consumption has been hypothesized to improve insulin sensitivity, thereby reducing the risk of diabetes and subsequently lowering the risk of liver cancer [30]. However, in populations with diabetes, metabolic disorders caused by impaired insulin secretion from β-cells likely make any level of alcohol consumption a risk factor for liver cancer [31]. Our findings reinforce that alcohol consumption, regardless of amount, is not recommended for cancer prevention, particularly in individuals with diabetes.

The epidemiological evidence for the effect of tea consumption on liver cancer risk remains inconclusive. While some meta-analyses have found no association between tea consumption and liver cancer risk [12,13], others suggested that green tea consumption may significantly reduce the risk [10,11]. Green tea, unlike other types, is produced by steaming or pan-firing fresh tea leaves at high temperatures, minimizing the oxidation of its primary polyphenols, particularly catechins [20]. The major catechin in green tea, epigallocatechin-3-gallate (EGCG), exhibits strong antioxidant properties and inhibits the initiation and progression of various cancers. However, EGCG can also act as pro-oxidant, and excessive consumption may even lead to hepatotoxicity [32]. In our study, among non-diabetic participants, modest tea consumption (low-level or <30 years) was associated with a reduced risk of liver cancer, but higher consumption did not confer additional protection. This may reflect the dual role of EGCG in cancer prevention and potential toxicity. Additionally, no protective effect was observed in diabetic participants, possibly due to the alterations in gut microbiota [33]. Gut microbiota transform tea polyphenols into bioactive metabolites, and further research is needed to determine whether gut microbiota changes in diabetic individuals affect the metabolism and efficacy of these compounds [34].

To better combat liver cancer risk in diabetic populations, it is important to reduce both the frequency and amount of alcohol consumption, particularly the consumption of high-proof spirits, and to limit the duration of drinking. Special attention should be given to the alcohol consumption behavior of diabetic patients who are middle-aged, male, living in rural areas, smokers, or overweight. According to the “Standards of medical care for type 2 diabetes in China”, alcohol consumption is not recommended for diabetic patients [35]. Additionally, female diabetic patients can reduce the risk of liver cancer by regularly consuming tea.

To the best of our knowledge, this is by far the largest prospective cohort study assessing the association between beverage consumption (alcohol and tea) and liver cancer secondary to diabetes. The strengths of our study included a large sample size of 0.5 million, coverage across 10 urban and rural regions in China, rich covariate data, and long-term follow-up. In addition to frequency of consumption, we measured the amount of consumption in grams of alcohol and tea leaves, which provided a more accurate intake compared with the estimated serving size used in previous studies. Furthermore, our study expanded the analysis by assessing the duration and types of consumption (e.g., spirits, green tea).

Our study has several limitations. First, the information on tea and alcohol consumption was self-reported, which might lead to some misclassification. Additionally, the consumption data were collected at baseline, potentially missing changes during follow-up. Second, we cannot exclude residual confounding by other unmeasured or unknown factors, such as coffee consumption. To the best of our knowledge, based on a re-survey involving approximately 5% of randomly selected surviving CKB participants, fewer than 2% of participants consumed coffee weekly [36]; therefore, such confounding should be minimum. Third, some cases of liver cancer during follow-up might not have been diagnosed, even though we linked inpatient records from the health insurance database and cancer data from local disease registries.

## 5. Conclusions

In conclusion, this large cohort study of Chinese adults indicated that alcohol consumption was associated with an increased risk of liver cancer, particularly among diabetic patients. Frequent, high-level, and long-term alcohol consumption were all strongly correlated with elevated risk. However, the protective effects of low-level tea and alcohol consumption observed in non-diabetic individuals were absent in the diabetic population, appearing only evident in certain populations, such as females and non-smokers. These findings highlight the importance of reducing alcohol consumption as a strategy for liver cancer prevention in the diabetic population, while also suggesting potential benefits of tea intake in specific subgroups.

## Figures and Tables

**Table 1:** Baseline characteristics of participants according to alcohol consumption after propensity score matching ^a^.

	Participants with Diabetes ^b^ (Cases/*n* = 193/30,289)	Alcohol Consumption			Participants Without Diabetes ^b^ (Cases/*n* = 398/90,867)	Alcohol Consumption		
		Less Than Weekly ^b^ (Cases/*n* = 144/26,549)	Weekly ^b^ (Cases/*n* = 49/3740)	*p* ^c^		Less Than Weekly ^b^ (Cases/*n* = 305/78,248)	Weekly ^b^ (Cases/*n* = 93/12,619)	*p* ^c^
Age	58.19 (9.58)	58.62 (9.46)	55.09 (9.90)	<0.001	58.19 (9.58)	58.46 (9.51)	56.50 (9.82)	<0.001
Sex				<0.001				<0.001
Male	11,678 (38.56%)	8173 (30.78%)	3505 (93.72%)		35,031 (38.55%)	23,822 (30.44%)	11,209 (88.83%)	
Female	18,611 (61.44%)	18,376 (69.22%)	235 (6.28%)		55,836 (61.45%)	54,426 (69.56%)	1410 (11.17%)	
Smoking				<0.001				<0.001
Never smoker	19,390 (64.02%)	18,902 (71.20%)	488 (13.05%)		57,162 (62.91%)	55,305 (70.68%)	1857 (14.72%)	
Occasional smoker	1573 (5.19%)	1302 (4.90%)	271 (7.25%)		4673 (5.14%)	3771 (4.82%)	902 (7.15%)	
Ex regular smoker	2855 (9.43%)	2183 (8.22%)	672 (17.97%)		6313 (6.95%)	4579 (5.85%)	1734 (13.74%)	
Smoker	6471 (21.36%)	4162 (15.68%)	2309 (61.74%)		22,719 (25.00%)	14,593 (18.65%)	8126 (64.39%)	
Meat intake				<0.001				<0.001
Daily	10,842 (35.80%)	8941 (33.68%)	1901 (50.83%)		24,658 (27.14%)	19,877 (25.40%)	4781 (37.89%)	
4–6 days per week	4875 (16.09%)	4230 (15.93%)	645 (17.25%)		15,957 (17.56%)	13,540 (17.30%)	2417 (19.15%)	
1–3 days per week	9952 (32.86%)	8930 (33.64%)	1022 (27.33%)		33,674 (37.06%)	29,081 (37.17%)	4593 (36.40%)	
Monthly	3209 (10.59%)	3067 (11.55%)	142 (3.80%)		11,825 (13.01%)	11,145 (14.24%)	680 (5.39%)	
Never/rarely	1411 (4.66%)	1381 (5.20%)	30 (0.80%)		4753 (5.23%)	4605 (5.89%)	148 (1.17%)	
Vegetable intake				0.021				<0.001
Daily	29,012 (95.78%)	25,392 (95.64%)	3620 (96.79%)		86,037 (94.68%)	73,815 (94.33%)	12,222 (96.85%)	
4–6 days per week	881 (2.91%)	801 (3.02%)	80 (2.14%)		3275 (3.60%)	3009 (3.85%)	266 (2.11%)	
1–3 days per week	343 (1.13%)	308 (1.16%)	35 (0.94%)		1263 (1.39%)	1148 (1.47%)	115 (0.91%)	
Monthly	42 (0.14%)	38 (0.14%)	4 (0.11%)		257 (0.28%)	246 (0.31%)	11 (0.09%)	
Never/rarely	11 (0.04%)	10 (0.04%)	1 (0.03%)		35 (0.04%)	30 (0.04%)	5 (0.04%)	
Fruits intake				<0.001				<0.001
Daily	6378 (21.06%)	5608 (21.12%)	770 (20.59%)		16,843 (18.54%)	14,757 (18.86%)	2086 (16.53%)	
4–6 days per week	2455 (8.11%)	2209 (8.32%)	246 (6.58%)		8003 (8.81%)	7089 (9.06%)	914 (7.24%)	
1–3 days per week	8640 (28.53%)	7477 (28.16%)	1163 (31.10%)		27,657 (30.44%)	23,579 (30.13%)	4078 (32.32%)	
Monthly	8969 (29.61%)	7921 (29.84%)	1048 (28.02%)		32,076 (35.30%)	27,777 (35.50%)	4299 (34.07%)	
Never/rarely	3847 (12.70%)	3334 (12.56%)	513 (13.72%)		6288 (6.92%)	5046 (6.45%)	1242 (9.84%)	
Physical activity (MET-h/d)	15.37 (11.85)	14.98 (11.58)	18.14 (13.27)	<0.001	18.40 (13.02)	17.99 (12.70)	20.94 (14.59)	<0.001
BMI	25.07 (3.59)	25.06 (3.61)	25.13 (3.43)	0.255	23.58 (3.46)	23.60 (3.50)	23.44 (3.22)	<0.001
Chronic liver disease				<0.001				0.833
No	29,150 (96.24%)	25,596 (96.41%)	3554 (95.03%)		87,673 (96.48%)	75,493 (96.48%)	12,180 (96.52%)	
Yes	1139 (3.76%)	953 (3.59%)	186 (4.97%)		3194 (3.52%)	2755 (3.52%)	439 (3.48%)	
Hypertension				<0.001				<0.001
No	21,447 (70.81%)	18,461 (69.54%)	2986 (79.84%)		77,129 (84.88%)	65,916 (84.24%)	11,213 (88.86%)	
Yes	8842 (29.19%)	8088 (30.46%)	754 (20.16%)		13,738 (15.12%)	12,332 (15.76%)	1406 (11.14%)	
Family history ^d^				<0.001				<0.001
No	20,922 (69.07%)	18,490 (69.64%)	2432 (65.03%)		72,197 (79.45%)	62,375 (79.71%)	9822 (77.84%)	
Yes	9367 (30.93%)	8059 (30.36%)	1308 (34.97%)		18,670 (20.55%)	15,873 (20.29%)	2797 (22.16%)	

Abbreviations: MET, metabolic equivalent of task; BMI: body mass index. ^a^ Propensity score matching (1:3) was performed by age and sex between diabetic and non-diabetic participants. ^b^ Values are presented as *n* (%) or as mean (SD). ^c^ *p* from Pearson’s Chi-squared test, Fisher’s exact test, or Welch’s two-sample *t*-test, depending on the data. ^d^ Diagnosed with cancer or diabetes in parents or siblings.

**Table 2:** Baseline characteristics of participants according to tea consumption after propensity score matching ^a^.

	Participants with Diabetes ^b^ (Cases/*n* = 193/30,289)	Tea Consumption				Participants Without Diabetes ^b^ (Cases/*n* = 398/90,867) ^d^	Tea Consumption			
		Never ^b^ (Cases/*n* = 72/12,067)	Less Than Weekly ^b,c^ (Cases/*n* = 50/8701)	Weekly ^b^ (Cases/*n* = 71/9521)	*p* ^d^		Never ^b^ (Cases/*n* = 145/35,399)	Less Than Weekly ^b,c^ (Cases/*n* = 106/25,931)	Weekly ^b^ (Cases/*n* = 147/29,537)	*p* ^d^
Age	58.19 (9.58)	59.37 (9.27)	57.14 (9.62)	57.63 (9.78)	<0.001	58.19 (9.58)	59.63 (9.24)	56.72 (9.64)	57.74 (9.69)	<0.001
Sex					<0.001					<0.001
Male	11,678 (38.56%)	2698 (22.36%)	3339 (38.37%)	5641 (59.25%)		35,031 (38.55%)	7534 (21.28%)	9581 (36.95%)	17,916 (60.66%)	
Female	18,611 (61.44%)	9369 (77.64%)	5362 (61.63%)	3880 (40.75%)		55,836 (61.45%)	27,865 (78.72%)	16,350 (63.05%)	11,621 (39.34%)	
Smoking					<0.001					<0.001
Never smoker	19,390 (64.02%)	9466 (78.45%)	5529 (63.54%)	4395 (46.16%)		57,162 (62.91%)	28,053 (79.25%)	16,424 (63.34%)	12,685 (42.95%)	
Occasional smoker	1573 (5.19%)	446 (3.70%)	625 (7.18%)	502 (5.27%)		4673 (5.14%)	1321 (3.73%)	1870 (7.21%)	1482 (5.02%)	
Ex regular smoker	2855 (9.43%)	870 (7.21%)	796 (9.15%)	1189 (12.49%)		6313 (6.95%)	1827 (5.16%)	1753 (6.76%)	2733 (9.25%)	
Smoker	6471 (21.36%)	1285 (10.65%)	1751 (20.12%)	3435 (36.08%)		22,719 (25.00%)	4198 (11.86%)	5884 (22.69%)	12,637 (42.78%)	
Meat intake					<0.001					<0.001
Daily	10,842 (35.80%)	3611 (29.92%)	3298 (37.90%)	3933 (41.31%)		24,658 (27.14%)	7933 (22.41%)	7842 (30.24%)	8883 (30.07%)	
4–6 days per week	4875 (16.09%)	1527 (12.65%)	1482 (17.03%)	1866 (19.60%)		15,957 (17.56%)	5091 (14.38%)	4659 (17.97%)	6207 (21.01%)	
1–3 days per week	9952 (32.86%)	4125 (34.18%)	2913 (33.48%)	2914 (30.61%)		33,674 (37.06%)	12,825 (36.23%)	9825 (37.89%)	11,024 (37.32%)	
Monthly	3209 (10.59%)	1853 (15.36%)	749 (8.61%)	607 (6.38%)		11,825 (13.01%)	6400 (18.08%)	2688 (10.37%)	2737 (9.27%)	
Never/rarely	1411 (4.66%)	951 (7.88%)	259 (2.98%)	201 (2.11%)		4753 (5.23%)	3150 (8.90%)	917 (3.54%)	686 (2.32%)	
Vegetable intake					0.177					<0.001
Daily	29,012 (95.78%)	11,537 (95.61%)	8349 (95.95%)	9126 (95.85%)		86,037 (94.68%)	33,283 (94.02%)	24,693 (95.23%)	28,061 (95.00%)	
4–6 days per week	881 (2.91%)	371 (3.07%)	247 (2.84%)	263 (2.76%)		3275 (3.60%)	1437 (4.06%)	861 (3.32%)	977 (3.31%)	
1–3 days per week	343 (1.13%)	141 (1.17%)	96 (1.10%)	106 (1.11%)		1263 (1.39%)	530 (1.50%)	323 (1.25%)	410 (1.39%)	
Monthly	42 (0.14%)	14 (0.12%)	6 (0.07%)	22 (0.23%)		257 (0.28%)	130 (0.37%)	46 (0.18%)	81 (0.27%)	
Never/rarely	11 (0.04%)	4 (0.03%)	3 (0.03%)	4 (0.04%)		35 (0.04%)	19 (0.05%)	8 (0.03%)	8 (0.03%)	
Fruits intake					<0.001					<0.001
Daily	6378 (21.06%)	2359 (19.55%)	1896 (21.79%)	2123 (22.30%)		16,843 (18.54%)	6314 (17.84%)	5338 (20.59%)	5191 (17.57%)	
4–6 days per week	2455 (8.11%)	806 (6.68%)	770 (8.85%)	879 (9.23%)		8003 (8.81%)	2769 (7.82%)	2550 (9.83%)	2684 (9.09%)	
1–3 days per week	8640 (28.53%)	3173 (26.29%)	2655 (30.51%)	2812 (29.53%)		27,657 (30.44%)	9969 (28.16%)	8210 (31.66%)	9478 (32.09%)	
Monthly	8969 (29.61%)	3762 (31.18%)	2427 (27.89%)	2780 (29.20%)		32,076 (35.30%)	13,273 (37.50%)	8355 (32.22%)	10,448 (35.37%)	
Never/rarely	3847 (12.70%)	1967 (16.30%)	953 (10.95%)	927 (9.74%)		6288 (6.92%)	3074 (8.68%)	1478 (5.70%)	1736 (5.88%)	
Physical activity (MET-h/d)	15.37 (11.85)	14.80 (11.65)	15.96 (11.96)	15.55 (11.97)	<0.001	18.40 (13.02)	17.77 (13.00)	19.16 (13.12)	18.50 (12.92)	<0.001
BMI	25.07 (3.59)	24.94 (3.63)	25.16 (3.54)	25.15 (3.58)	<0.001	23.58 (3.46)	23.61 (3.51)	23.77 (3.37)	23.37 (3.47)	<0.001
Chronic liver disease					0.043					0.131
No	29,150 (96.24%)	11,650 (96.54%)	8370 (96.20%)	9130 (95.89%)		87,673 (96.48%)	34,129 (96.41%)	25,070 (96.68%)	28,474 (96.40%)	
Yes	1139 (3.76%)	417 (3.46%)	331 (3.80%)	391 (4.11%)		3194 (3.52%)	1270 (3.59%)	861 (3.32%)	1063 (3.60%)	
Hypertension					<0.001					<0.001
No	21,447 (70.81%)	8343 (69.14%)	6325 (72.69%)	6779 (71.20%)		77,129 (84.88%)	29,657 (83.78%)	22,412 (86.43%)	25,060 (84.84%)	
Yes	8842 (29.19%)	3724 (30.86%)	2376 (27.31%)	2742 (28.80%)		13,738 (15.12%)	5742 (16.22%)	3519 (13.57%)	4477 (15.16%)	
Family history ^e^					<0.001					<0.001
No	20,922 (69.07%)	8306 (68.83%)	5909 (67.91%)	6707 (70.44%)		72,197 (79.45%)	27,766 (78.44%)	20,422 (78.76%)	24,009 (81.28%)	
Yes	9367 (30.93%)	3761 (31.17%)	2792 (32.09%)	2814 (29.56%)		18,670 (20.55%)	7633 (21.56%)	5509 (21.24%)	5528 (18.72%)	

Abbreviations: MET, metabolic equivalent of task; BMI: body mass index. ^a^ Propensity score matching (1:3) was performed by age and sex between diabetic and non-diabetic participants. ^b^ Values are presented as *n* (%) or as mean (SD). ^c^ Those who never drink tea were not included. ^d^ *p* from Pearson’s Chi-squared test, Fisher’s exact test, or ANOVA, depending on the data. ^e^ Diagnosed with cancer or diabetes in parents or siblings.

**Table 3:** Hazard ratios for liver cancer incidence according to duration and types of beverage consumption ^a^.

	Diabetic			Non-Diabetic		
	Cases	Cases/PYs (/1000)	HR (95% CI)	Cases	Cases/PYs (/1000)	HR (95% CI)
Alcohol consumption (*n* = 512,581)						
Duration of alcohol consumption						
Less than weekly	144	0.58	Reference	1300	0.32	Reference
<10 years	6	1.32	1.52 (0.66 to 3.50)	55	0.40	1.02 (0.78 to 1.35)
10–29 years	19	1.10	1.57 (0.93 to 2.65)	179	0.47	1.12 (0.95 to 1.32)
≥30 years	24	1.81	1.70 (1.06 to 2.71)	209	1.02	1.35 (1.16 to 1.58)
Types of alcohol consumed						
Less than weekly	144	0.58	Reference	1300	0.32	Reference
Beer (small and/or large)	8	0.95	1.53 (0.72 to 3.25)	43	0.32	0.91 (0.67 to 1.24)
Rice wine/Wine	7	1.59	1.42 (0.65 to 3.12)	56	0.62	0.99(0.75 to 1.30)
Spirit (<50% alcohol)	9	1.31	1.29 (0.64 to 2.60)	124	0.77	1.42 (1.17 to 1.72)
Spirit (≥50% alcohol)	25	1.61	1.91 (1.20 to 3.04)	220	0.65	1.24 (1.07 to 1.45)
Tea consumption (*n* = 352,890)						
Duration of tea consumption						
Never	72	0.64	Reference	548	0.32	Reference
<10 years	15	0.81	1.11 (0.63 to 1.98)	87	0.29	0.74 (0.58 to 0.93)
10–29 years	23	0.63	0.71 (0.43 to 1.19)	261	0.34	0.84 (0.71 to 0.99)
≥30 years	33	0.99	0.87 (0.55 to 1.37)	333	0.62	0.95 (0.81 to 1.10)
Types of tea consumed						
Never	72	0.64	Reference	548	0.32	Reference
Green tea	60	0.80	0.87 (0.60 to 1.27)	549	0.40	0.86 (0.75 to 0.98)
Others	11	0.79	0.78 (0.40 to 1.53)	132	0.55	0.94 (0.76 to 1.16)

Abbreviations: CI, confidence interval; HR, hazard ratio; PYs, person years. ^a^ Adjusted for age, sex, region, education, marital status, household income, smoking, meat intake, vegetable intake, fruit intake, physical activity, BMI, chronic liver disease, hypertension, and family history.

## Data Availability

Details on how to access China Kadoorie Biobank data and what data are currently available to open access users are available from https://www.ckbiobank.org/data-access (accessed on 1 August 2025).

## Supplementary Materials

The following supporting information can be downloaded at: https://www.mdpi.com/article/10.3390/nu17172870/s1, Figure S1: Flow chart of participants inclusion process; Figure S2: Kaplan–Meier curves of liver cancer incidence stratified by alcohol and tea consumption in diabetic and non-diabetic participants; Figure S3: Relationship of weekly green tea intake and duration with liver cancer incidence in participants without diabetes; Table S1: Interaction analyses of diabetes with alcohol and tea consumption on liver cancer risk; Table S2: Subgroup analysis of associations between alcohol consumption and risk of liver cancer incidence in participants with diabetes according to potential baseline risk factors (*n* = 30,289); Table S3: Subgroup analysis of associations between tea consumption and risk of liver cancer incidence in participants with diabetes according to potential baseline risk factors (*n* = 30,289); Table S4: Sensitivity analysis of associations between alcohol consumption and risk of liver cancer incidence in participants with diabetes (*n* = 30,289); Table S5: Sensitivity analysis of associations between tea consumption and risk of liver cancer incidence in participants with diabetes (*n* = 30,289).

## Abbreviations

The following abbreviations are used in this manuscript:

AP	Proportion Attributable to Interaction
BMI	Body Mass Index
CI	Confidence Interval
CKB	China Kadoorie Biobank
EGCG	Epigallocatechin-3-Gallate
HR	Hazard Ratio
ICD-10	International Classification of Diseases, 10th Revision
IGF-I	Insulin-like Growth Factor I
MET	Metabolic Equivalent of Task
PSM	Propensity Score Matching
PY	Person Year
RCS	Restricted Cubic Splines
RERI	Relative Excess Risk Due to Interaction
S	Synergy Index
SD	Standard Deviation
