# Supplementary material for: Alcohol and Tea Consumption in Relation to Liver Cancer Risk by Diabetes Status: A Prospective Cohort Study of 0.5 Million Chinese Adults
Source: Nutrients. 2025 Sep 4;17(17):2870. doi: 10.3390/nu17172870 (PMC12430480; doi:10.3390/nu17172870)
Supplement: Supplementary file 1 [file nutrients-17-02870-s001.zip › nutrients-3831420-supplementary.pdf]

Supplementary Materials

Alcohol and Tea Consumption in Relation to Liver Cancer Risk by Diabetes Status: A Prospective Cohort Study of 0.5 Million Chinese Adults

Xiaoru Feng, Ruoqian Li, Minqing Yan, Changzheng Yuan, You Wu*
* Corresponding author: School of Healthcare Management, Tsinghua Medicine, Tsinghua University (youwu@tsinghua.edu.cn)

Table of Contents

Figure S1. Flow chart of participants inclusion process. **2**

Figure S2. Kaplan-Meier curves of liver cancer incidence stratified by alcohol and tea consumption in diabetic and non-diabetic participants. **3**

Figure S3. Relationship of weekly green tea intake and duration with liver cancer incidence in participants without diabetes. **4**

Table S1. Interaction analyses of diabetes with alcohol and tea consumption on liver cancer risk. **5**

Table S2. Subgroup analysis of associations between alcohol consumption and risk of liver cancer incidence in participants with diabetes according to potential baseline risk factors (*n*=30,289). **6**

Table S3. Subgroup analysis of associations between tea consumption and risk of liver cancer incidence in participants with diabetes according to potential baseline risk factors (*n*=30,289). **7**

Table S4. Sensitivity analysis of associations between alcohol consumption and risk of liver cancer incidence in participants with diabetes (*n*=30,289). **8**

Table S5. Sensitivity analysis of associations between tea consumption and risk of liver cancer incidence in participants with diabetes (*n*=30,289). **9**

Figure S1. Flow chart of participants inclusion process.

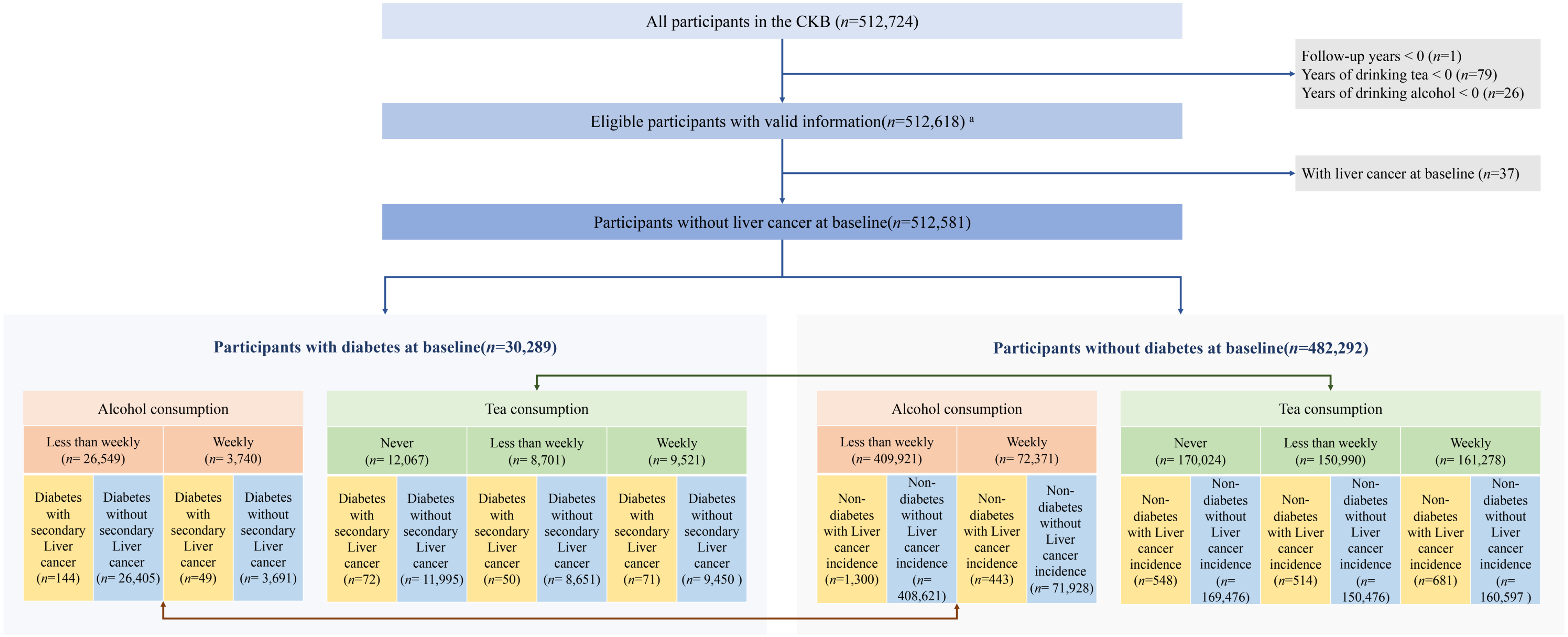

[a] Missing values in weight (n = 2) were imputed using the median value.

Figure S2. Kaplan-Meier curves of liver cancer incidence stratified by alcohol and tea consumption in diabetic and non-diabetic participants.

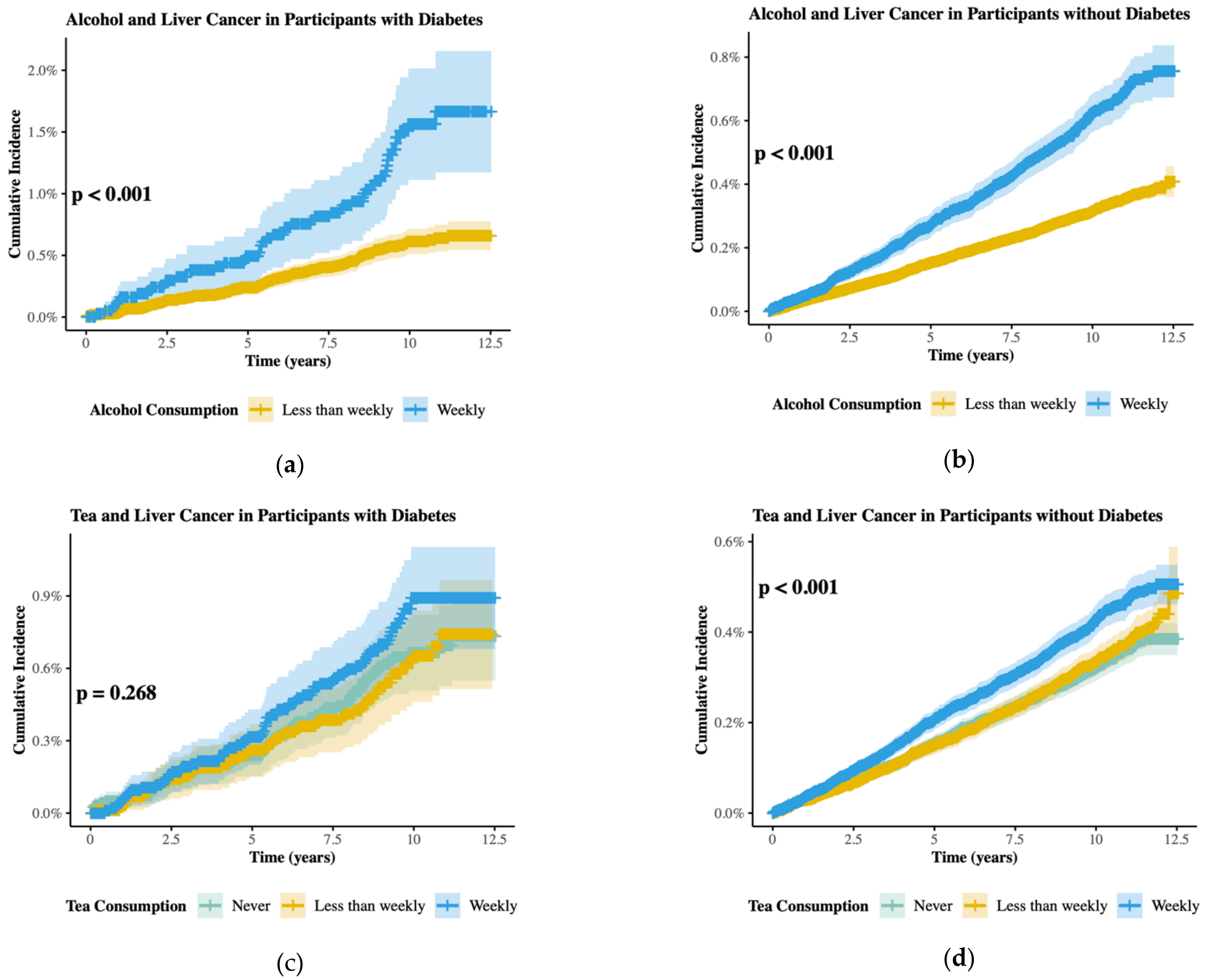

P-values were calculated by the log-rank test, and Kaplan–Meier curves are presented without multivariable adjustment. (a) Kaplan-Meier curve of liver cancer incidence by alcohol consumption in diabetic participants; (b) Kaplan-Meier curve of liver cancer incidence by alcohol consumption in non-diabetic participants; (c) Kaplan-Meier curve of liver cancer incidence by tea consumption in diabetic participants; (d) Kaplan-Meier curve of liver cancer incidence by tea consumption in non-diabetic participants.

Figure S3. Relationship of weekly green tea intake and duration with liver cancer incidence in participants without diabetes.

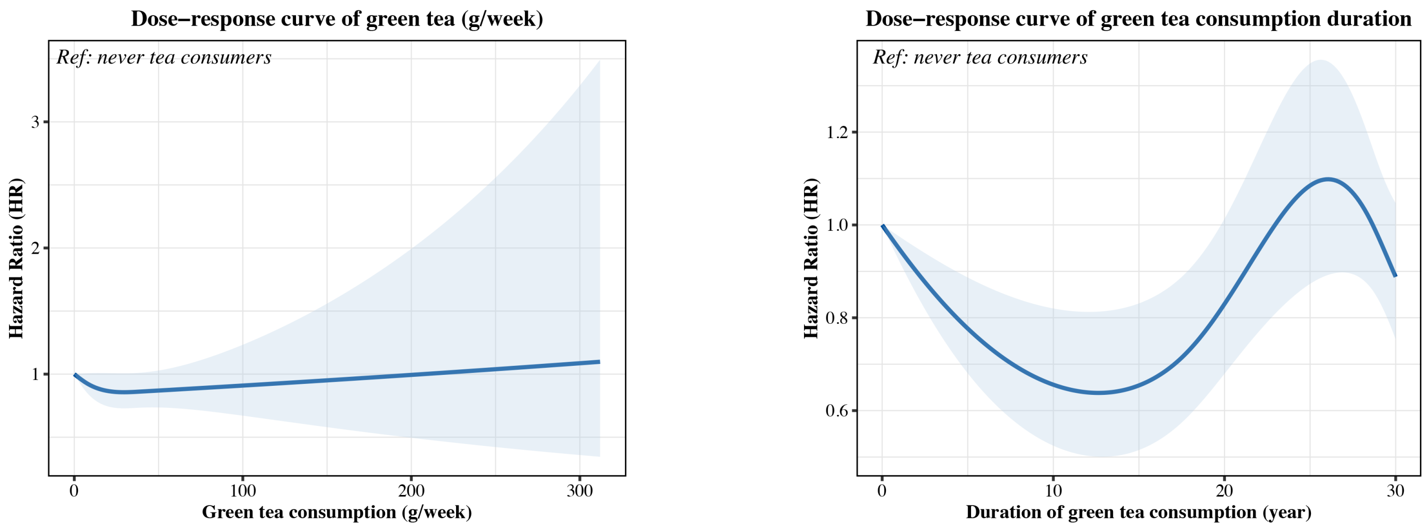

(a) (b)

(a) Relationship of weekly green tea intake (grams) with liver cancer incidence in participants without diabetes；(b) Relationship of weekly green tea consumption duration (years) with liver cancer incidence in participants without diabetes.

Table S1. Interaction analyses of diabetes with alcohol and tea consumption on liver cancer risk.

	RR	RERI	AP	S
**Alcohol consumption**				
No diabetes & Less than weekly	Reference			
Diabetes & Less than weekly	1.46	0.56	0.25	1.84
No diabetes & Weekly	1.21			
Diabetes & Weekly	2.23			
**Tea consumption**				
No diabetes & Consumption [a]	Reference			
Diabetes & Consumption	1.60			
No diabetes & Never	0.96	0.13	0.08	1.25
Diabetes & Never	1.44			

Abbreviations: RERI, relative excess risk due to interaction; AP, proportion attributable to interaction; S, synergy index

[a] "Consumption" refers to all tea consumers excluding those who never drank tea.

Table S2. Subgroup analysis of associations between alcohol consumption and risk of liver cancer incidence in participants with diabetes according to potential baseline risk factors (*n*=30,289). [a]

	Less than weekly			Weekly			$P_{interaction}$ [b]
	Cases	Cases/PYs (/1000)	HR	Cases	Cases/PYs (/1000)	HR (95% CI)	
Age (years)							0.505
<55	34	0.37	1	15	0.78	1.39 (0.70, 2.76)	
55-65	55	0.62	1	22	2.26	1.88 (1.08, 3.26)	
≥65	55	0.84	1	12	1.95	1.50 (0.75, 2.98)	
Sex							0.548
Male	74	1.01	1	46	1.40	1.50 (1.02, 2.19)	
Female	70	0.40	1	3	1.36	2.47 (0.74, 8.23)	
Region							0.925
Rural	56	0.56	1	17	1.67	1.98 (1.08, 3.63)	
Urban	88	0.59	1	32	1.28	1.53 (0.97, 2.42)	
Smoking							0.834
Never smoker	72	0.40	1	4	0.86	1.96 (0.67, 5.71)	
Smoker [c]	72	1.06	1	45	1.47	1.53 (1.04, 2.24)	
Physical activity (MET h/day)							0.956
Male<5.66/Female<8.40	35	0.64	1	10	1.68	1.24 (0.57, 2.67)	
Male 5.66-12.19/Female8.40-11.69	43	0.65	1	14	1.92	1.66 (0.84, 3.27)	
Male12.20-23.44/Female11.70-18.19	39	0.62	1	13	1.23	1.95 (0.94, 4.06)	
Male≥23.45/Female≥18.20	27	0.43	1	12	1.06	1.33 (0.62, 2.85)	
BMI (kg/m^2)							0.278
<25.0	80	0.64	1	23	1.37	1.31 (0.79, 2.17)	
≥25.0	64	0.52	1	26	1.42	1.97 (1.16, 3.35)	

Abbreviations: PYs, person years; HR, hazard ratio; CI, confidence interval; MET, metabolic equivalent of task (grouped by quartiles according to sex); BMI: body mass index.

[a] Except for the baseline stratifying variable, the model was adjusted for the same variables as in the main analysis.

[b] *P* from the likelihood ratio tests for interaction.

[c] "Occasional smoker" and "Ex-regular smoker" were categorized into "smoker."

Table S3. Subgroup analysis of associations between tea consumption and risk of liver cancer incidence in participants with diabetes according to potential baseline risk factors (*n*=30,289). [a]

	Never			Less than weekly [b]			Weekly			$P_{interaction}$ [c]
	Cases	Cases/PYs (/1000)	HR	Cases	Cases/PYs (/1000)	HR (95% CI)	Cases	Cases/PYs (/1000)	HR (95% CI)	
Age (years)										0.617
<55	15	0.39	1	16	0.45	0.87 (0.42, 1.81)	18	0.47	0.71 (0.34, 1.50)	
55-65	25	0.60	1	21	0.75	0.85 (0.47, 1.54)	31	1.07	0.99 (0.57, 1.74)	
≥65	32	1.00	1	13	0.71	0.61 (0.32, 1.18)	22	1.01	0.77 (0.43, 1.38)	
Sex										0.011
Male	24	1.02	1	32	1.05	1.06 (0.62, 1.82)	64	1.23	1.31 (0.81, 2.11)	
Female	48	0.54	1	18	0.35	0.72 (0.42, 1.26)	7	0.19	0.36 (0.16, 0.81)	
Region										0.583
Rural	26	0.57	1	21	0.69	1.13 (0.63, 2.05)	26	0.78	1.11 (0.61, 2.03)	
Urban	46	0.69	1	29	0.57	0.66 (0.41, 1.07)	45	0.81	0.78 (0.50, 1.22)	
Smoking										0.055
Never smoker	46	0.52	1	19	0.36	0.76 (0.44, 1.32)	11	0.27	0.49 (0.25, 0.97)	
Smoker [d]	26	1.15	1	31	1.07	0.92 (0.54, 1.56)	60	1.27	1.17 (0.73, 1.88)	
Physical activity (MET h/day)										0.867
Male<5.66/Female<8.40	19	0.73	1	12	0.73	0.74 (0.35, 1.56)	14	0.77	0.72 (0.35, 1.50)	
Male 5.66-12.19/Female8.40-11.69	22	0.74	1	14	0.70	0.84 (0.42, 1.68)	21	0.90	0.86 (0.44, 1.65)	
Male12.20-23.44/Female11.70-18.19	20	0.73	1	11	0.51	0.59 (0.27, 1.25)	21	0.85	0.89 (0.46, 1.75)	
Male≥23.45/Female≥18.20	11	0.38	1	13	0.56	1.23 (0.54, 2.84)	15	0.68	1.05 (0.45, 2.48)	
BMI (kg/m^2)										0.045
<25.0	43	0.74	1	19	0.47	0.56 (0.32, 0.97)	41	0.96	0.94 (0.59, 1.50)	
≥25.0	29	0.54	1	31	0.75	1.17 (0.7, 1.98)	30	0.66	0.83 (0.48, 1.42)	

Abbreviations: PYs, person years; HR, hazard ratio; CI, confidence interval; MET, metabolic equivalent of task (grouped by quartiles according to sex); BMI: body mass index.

[a] Except for the baseline stratifying variable, the model was adjusted for the same variables as in the main analysis.

[b] Those who never drink tea were not included.

[c] *P* from the likelihood ratio tests for interaction.

[d] "Occasional smoker" and "Ex-regular smoker" were categorized into "smoker."

Table S4. Sensitivity analysis of associations between alcohol consumption and risk of liver cancer incidence in participants with diabetes (*n*=30,289). [a]

	Less than weekly	Weekly (all)	Weekly consumption [b]		
			Low	Moderate	High
**Excluding cases identified during the first 2 years of follow-up**					
Cases	118	41	8	19	14
Person years	247,043	35,148	9,249	17,123	8,776
Cases/PYs (/1000)	0.48	1.17	0.86	1.11	1.60
HR (95%CI)	1	1.79 (1.19,2.69)	1.30 (0.62,2.73)	1.78 (1.05,3.04)	2.34 (1.28,4.27)
**Excluding participants with prevalent comorbidity at baseline** [c]					
Cases	103	42	7	19	16
Person years	192,339	30,063	7,514	14,544	8,005
Cases/PYs (/1000)	0.54	1.40	0.93	1.31	2.00
HR (95%CI)	1	1.58 (1.05,2.37)	1.03 (0.47,2.27)	1.49 (0.88,2.53)	2.32 (1.31,4.13)
**Incorporating regional HBV prevalence as a contextual covariate**					
Cases	144	49	9	23	17
Person years	247,071	35,156	9,249	17,128	8,779
Cases/PYs (/1000)	0.58	1.39	0.97	1.34	1.94
HR (95%CI)	1	1.58 (1.05,2.37)	1.03 (0.47,2.27)	1.49 (0.88,2.53)	2.32 (1.31,4.13)

Abbreviations: CI, confidence interval; HR, hazard ratio; PYs, person years.

[a] Models were adjusted for the same variables as in the main analysis.

[b] Classified by quartiles: Low (male: ≤110g/week; female: ≤30g/week); Moderate (male: 110-407g/week; female: 30-135g/week); High (male: >407g/week; female: >135g/week).

[c] Participants with any of the following diagnoses were excluded from the analysis: any cancer, chronic heart disease, stroke or transient ischemic attack, gallstone or gallbladder disease, kidney disease.

Table S5. Sensitivity analysis of associations between tea consumption and risk of liver cancer incidence in participants with diabetes (*n*=30,289). [a]

	Never	Less than weekly [b]	Weekly (all)	Weekly consumption [c]		
				Low	Moderate	High
**Excluding cases identified during the first 2 years of follow-up**						
Cases	58	40	61	16	33	12
Person years	111,847	81,661	88,684	20,496	47,013	21,175
Cases/PYs (/1000)	0.52	0.49	0.69	0.78	0.70	0.57
HR (95%CI)	1	0.82 (0.54,1.25)	0.98 (0.66,1.45)	1.25 (0.71,2.21)	0.99 (0.63,1.56)	0.69 (0.36,1.33)
**Excluding participants with prevalent comorbidity at baseline [d]**						
Cases	47	39	59	17	30	12
Person years	84,223	65,662	72,516	16,391	38,812	17,313
Cases/PYs (/1000)	0.56	0.59	0.81	1.04	0.77	0.69
HR (95%CI)	1	0.82 (0.53,1.28)	0.86 (0.57,1.31)	1.35 (0.76,2.39)	0.80 (0.49,1.30)	0.62 (0.32,1.21)
**Incorporating regional HBV prevalence as a contextual covariate**						
Cases	72	50	71	19	38	14
Person years	111,861	81,673	88,693	20,498	47,018	21,177
Cases/PYs (/1000)	0.64	0.61	0.80	0.93	0.81	0.66
HR (95%CI)	1	0.80 (0.55,1.16)	0.86 (0.60,1.23)	1.16 (0.69,1.95)	0.86 (0.57,1.31)	0.61 (0.33,1.11)

Abbreviations: CI, confidence interval; HR, hazard ratio; PYs, person years.

[a] Models were adjusted for the same variables as in the main analysis.

[b] Those who never drink tea were not included.

[c] Classified by quartiles: Low (≤12g/week); Moderate (12-26g/week); High (>26g/week).

[d] Participants with any of the following diagnoses were excluded from the analysis: any cancer, chronic heart disease, stroke or transient ischemic attack, gallstone or gallbladder disease, kidney disease.
